# Synthesis and evaluation of properties of *N*,*N*-bis(perfluorooctyl)imine acetate sodium as a gas-wetting alteration agent

**DOI:** 10.1039/c7ra10742k

**Published:** 2018-02-20

**Authors:** Yanling Wang, Yongfei Li, Qian Wang, Qiang Li, Yue Zhang, Lin Yuan

**Affiliations:** College of Petroleum Engineering, China University of Petroleum (East China) Qingdao 266580 Shandong P. R. China wangyl@upc.edu.cn; College of Chemical Engineering, China University of Petroleum (East China) Qingdao 266580 Shandong P. R. China

## Abstract

The wettability of a rock’s surface is a vital factor for gas flow and fracturing fluid backflow. As a result of this, novel and effective gas wetting alteration agents are required. In this work, a gas-wetting alteration agent, *N*,*N*-bis(perfluorooctyl)imine acetate sodium, was synthesized and characterized by different methods. The wettability of a rock’s surface was evaluated by contact angle and imbibition measurements, the Owens two-liquid method and glass capillary tube rise testing. The results showed that after treatment with 0.5 wt% *N*,*N*-bis(perfluorooctyl)imine acetate sodium the contact angles of water and *n*-hexadecane on the surface of the rock increased from 36° and 0° to 140° and 119°, respectively. The surface free energy rapidly reduced from primeval 72 mN m^−1^ to 3.4 mN m^−1^ after treatment with 0.3 wt% *N*,*N*-bis(perfluorooctyl)imine acetate sodium. These values agreed with the imbibition measurements and the results of the glass capillary tube rise testing. Moreover, analysis by scanning electron microscopy (SEM) and energy dispersive spectroscopy (EDS) showed that the roughness of the rock surface significantly increased. The above results fully proved that the wettability of the rock surface is altered from its original water-wetting or oil-wetting to gas-wetting. Furthermore, thermal analysis demonstrated that the gas-wetting alteration agent has good thermal stability, which indicates its great potential to be used as a gas-wetting alteration agent for unconventional gas reservoirs under high temperature conditions.

## Abstract 2

A gas-wetting alteration agent, *N*,*N*-bis(perfluorooctyl)imine acetate sodium, was synthesized and characterized by different methods and the wettability of a rock surface was evaluated.

## Graphical abstract

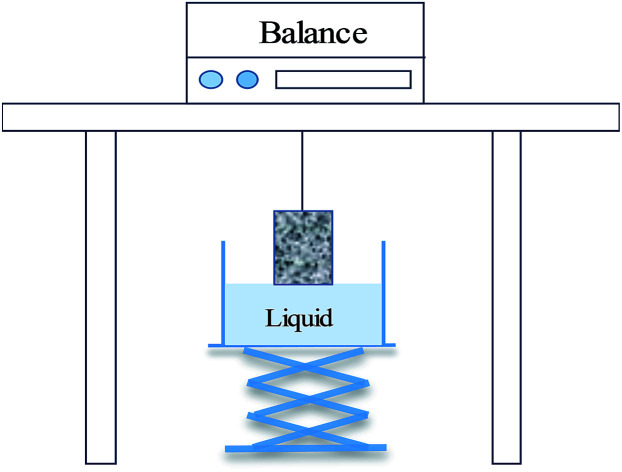

## 1. Introduction

With the change in energy patterns, shale gas has recently emerged as a relatively important energy source that provides an opportunity for a lot of countries around the world to reduce their dependence on energy imports and their struggle to achieve energy independence.^1^ It may also be a potential transition fuel that will permit the transformation from non-renewable to renewable energy resources as it is conducive to reducing emissions of carbon dioxide and other pollutants.^2^ However, shale gas is generally extracted from low-permeability or tight porous media. It cannot be captured from original source rock without an extra driving force.^3^ Hydraulic fracturing is the technology classically used to increase the production of shale gas,^4^ but this method is characterized by large displacement of liquid volume. Thus, fracturing fluid flowback plays a very important role in hydraulic fracturing operations. Its purpose is to promote the outflow of extra liquid which maintains the crevice with strong gas flow and to reduce damage to the formation.

Reservoir wettability is a crucial factor that dominates the distribution and flow of gas in shale media. It also has a remarkable influence on both the re-flow of fracturing fluid and the relative permeability of the reservoir.^5,6^ Thus, the investigation of reservoir wettability is of profound significance for improving shale gas recovery. Li *et al.*^7^ put forward the gas-wetting concept in which the wettability of rock was altered from liquid-wetting to neutral using a fluorocarbon surfactant. Tang and Firoozabadi^8^ demonstrated the altering of the wettability of sandstone from strong liquid-wetting to neutral gas-wetting using polymers. Feng *et al.*^9^ achieved the gas wetting alteration of a rock’s surface with organosilicon-acrylic and significantly improved gas production. Jin and Wang^10^ investigated the concept that the wettability of low-permeability rock could be permanently altered from strong water-wetting to intermediate by a novel gas-wetting alteration agent. Furthermore, Al-Yaseri *et al.*^11^ verified that the density of gas in the gas–liquid–solid system has a significant effect on the wettability. Iglauer *et al.*^12,13^ reported that gas-wetting increases with increasing pressure, salinity and dissolved ion valencies, while the effect of temperature is not well understood. Moreover, the influence of various subsurface minerals and rock types on the wettability and the mechanism of interaction were also investigated.

However, the hydrophobic and oleophobic groups of the gas-wetting alternation agents used in the previous studies were single-chain structures that cannot effectively alter the wettability of rock surfaces from liquid-wetting to gas-wetting. The objective of this study is to synthesize a gas-wetting alteration agent with a double-chain hydrophobic and oleophobic structure. The product can more effectively alter the reservoir wettability and improve the flowback rate of the fracturing fluid.

## 2. Experimental

### 2.1 Materials

Perfluorooctanoyl chloride was provided by Aladdin Industrial Corporation. Triethylamine was offered by Shandong Chemical Reagents Co., Ltd. Dichloromethane and sodium chloroacetate were supplied by Sinopharm Chemical Reagent Co. Ltd. Anhydrous ethanol and acetone were purchased from Shanghai Han Guang Chemical Reagent Co., Ltd. All of the above chemicals are analytical reagents. The rock samples used in this study were obtained from the Shengli Oilfield, China, and the basic parameters are shown in Table 1. During the experiment, the rock samples were polished using sandpaper to get a relatively smooth surface, then washed with 1.0 wt% NaCl solution and dried at 80 °C for 12 h.^14^ Deionized water was prepared in the laboratory.

**Table 1:** The basic parameters of rock

Type	Porosity (%)	Permeability (μm^2^)	Composition
Shale	4.13	11.8 × 10^−5^	Clay (>40%), quartz (>35%), carbonate (<20%)

### 2.2 Synthesis of the gas-wetting alteration agent

**Scheme 1:**
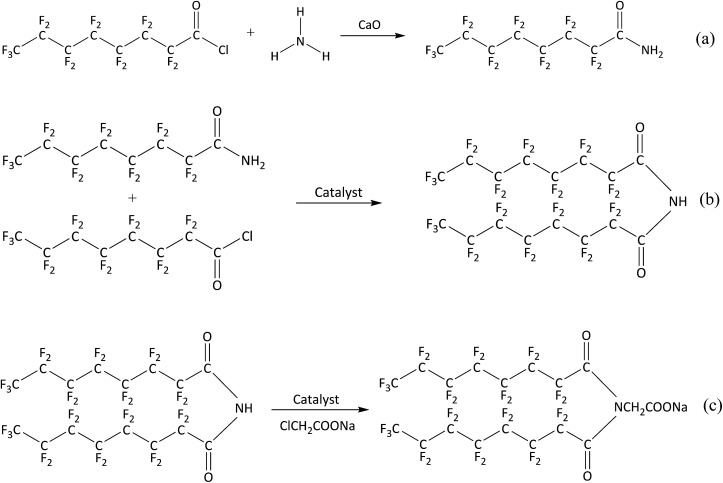
Synthesis of the gas-wetting alteration agent.

The gas-wetting alteration agent was synthesized according to the following procedures:

Firstly, perfluorooctanoyl chloride and calcium oxide were added to a flask in a molar ratio of 2 : 1 under a nitrogen atmosphere, and then anhydrous ethanol was added as solvent. Then, ammonia (a 3-fold molar excess of perfluorooctanoyl chloride) was injected and stirred for 4 h. The mixture was filtered under reduced pressure and dried in a universal oven at 78 °C to obtain perfluorooctanoyl ammonia. This first reaction is described in Scheme 1(a).

Secondly, 0.1 mol of perfluorooctanoyl ammonia was placed in a three-necked flask with anhydrous ethanol and triethylamine as the solvent and catalyst, respectively. Then, 0.1 mol of perfluorooctanoyl chloride was added dropwise under stirring and the temperature was kept below 5 °C for 6 h. *N*,*N*-bis(perfluorooctyl)imine was produced after removing the solvent and impurities. This procedure is shown in Scheme 1(b).

Finally, *N*,*N*-bis(perfluorooctyl)imine was added into a solution of acetone in a three-necked flask and sufficiently dissolved at 60 °C. Next, a solution of sodium chloroacetate was added dropwise to the mixture and vigorously stirred at room temperature for 6 h. The reaction mixture was filtered and concentrated under reduced pressure. Then, the residue was purified by recrystallization after removing the solvent. The purified material was steamed under reduced pressure to give the final product *N*,*N*-bis(perfluorooctyl)imine acetate sodium. This reaction is shown in Scheme 1(c).

### 2.3 Characterization

An FT-IR spectrum of the sample was obtained using a Bruker VERTEX 70 series Fourier transform infrared spectrometer in the wavenumber range of 4000–500 cm^−1^; ^1^H NMR spectra were recorded in the stated solutions, on a Bruker DRX-400 spectrometer, operating at 400 MHz for H; the thermal stability of the compound was analyzed using a TGA/SDTA851 thermal gravimetric analyzer with a heating rate of 10 °C min^−1^. The morphology and elements of the rock surface before and after treatment were examined by a JEOL JSM-6390A scanning electron microscope and energy dispersive spectrometer (EDAX Ltd., USA).

### 2.4 Methods

#### 2.4.1 Contact angle measurement

In order to measure the contact angle of water or *n*-hexadecane drops on original and treated rock surfaces, we used a micropipette to place a liquid drop on the surface of air-saturated rock at room temperature. The morphology of a sessile droplet on the rock surface was enlarged on a monitor screen. Pictures of the droplets were taken using a digital camera under proper illumination of a light source. The contact angle of the gas–liquid–rock three-phase system was measured using the software’s goniometry tool.^15^

#### 2.4.2 The Owens two-liquid method

There is a close relationship between the surface free energy and the wettability of a rock surface. In order to investigate the wettability alteration of the rock, its surface free energy was studied using the Owens two-liquid method.^16^ The surface free energy consists of the dispersion force and the polar force and the specific calculation equation is as follows:^17^

1 *γ*_s_ = *γ*^D^_s_ + *γ*^P^_s_

2 *γ*_L_(1 + cos *θ*) = 2(*γ*^D^_s_*γ*^D^_L_)^1/2^ + 2(*γ*^P^_s_*γ*^P^_L_)^1/2^

In eqn (1) and (2), *γ*_s_ is the surface free energy of the solid; *γ*^D^_s_ and *γ*^P^_s_ are the dispersion force and polar force of the solid, respectively; *γ*_L_ is the surface free energy of liquid; *θ* is the contact angle; *γ*^D^_L_ and *γ*^P^_L_ are the dispersion force and polar force of the liquid, respectively.

Variables *γ*^D^_s_ and *γ*^P^_s_ can be calculated using eqn (3) and (4).^18^

3 *γ*_L1_(1 + cos *θ*_1_) = 2(*γ*^D^_s_*γ*^D^_L1_)^1/2^ + 2(*γ*^P^_s_*γ*^P^_L1_)^1/2^

4 *γ*_L2_(1 + cos *θ*_2_) = 2(*γ*^D^_s_*γ*^D^_L2_)^1/2^ + 2(*γ*^P^_s_*γ*^P^_L2_)^1/2^

In eqn (3) and (4), *γ*_L1_ is the surface free energy of water; *θ*_1_ is the contact angle of water on the rock surface; *γ*^D^_L1_ and *γ*^P^_L1_ are the dispersion force and polar force of water, respectively; *γ*_L2_ is the surface free energy of *n*-hexadecane; *θ*_2_ is the contact angle of *n*-hexadecane on the rock surface; *γ*^D^_L2_ and *γ*^P^_L2_ are the dispersion force and polar force of *n*-hexadecane, respectively.

#### 2.4.3 Imbibition

**Fig. 1:**
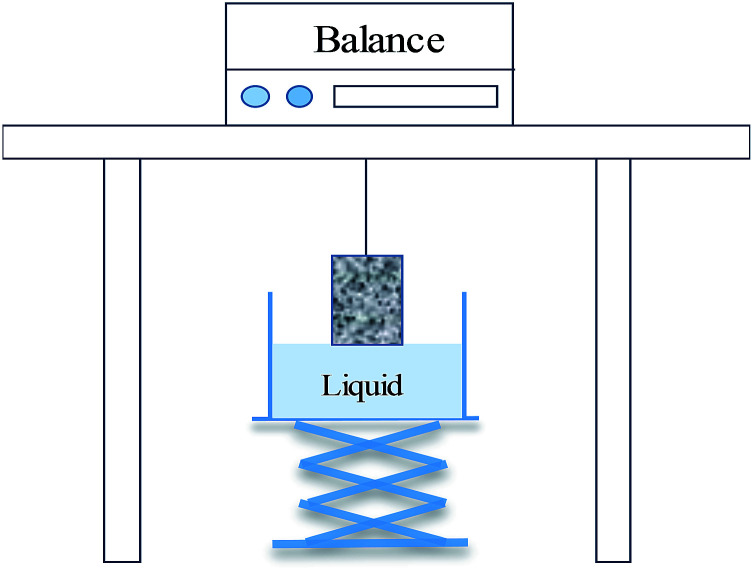
The simple device for spontaneous imbibition testing.

A spontaneous imbibition test can be conducted to evaluate the effect of wettability on fluid imbibed into the rock. The amount of liquid imbibed *versus* time was recorded using a balance when the bottom of rock touched the liquid surface.^19,20^ The readability of the balance was 0.001 g. A schematic showing the spontaneous imbibition test is exhibited in Fig. 1.

#### 2.4.4 The glass capillary tube rise test

**Fig. 2:**
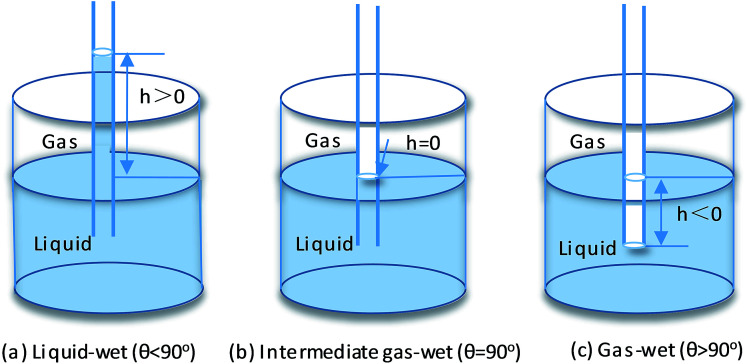
The simple device for glass capillary tube rise testing.

The glass capillary tube rise test was performed to verify the effect of the gas-wetting alteration agent on wettability alteration. The capillary radius used in this study was about 0.5 mm. Firstly, the glass capillary tube was washed with distilled water and dried at high temperature. Then, the cleaned glass capillaries were treated in a gas-wetting alteration agent solution for 24 h and dried at a certain temperature to remove excess solution from the tube. Finally, the treated glass capillaries were inserted into the solution. During the process, the liquid was sucked into the tube by capillary force. A schematic diagram is shown in Fig. 2. If the liquid was positive in the capillary (*h* > 0), the contact angle of the liquid on the rock surface was less than 90 °; when the liquid was neutral in the tube (*h* = 0), the contact angle was equal to 90 °; and if the liquid was negative (*h* < 0), then the contact angle of liquid was greater than 90 °. The specific contact angle in the gas–liquid–rock system could be computed using eqn (5), where *σ* and *ρ* represent the surface tension and density of the liquid, respectively; *r* is the radius of the glass capillary; *θ* is the contact angle; *h* is the height of the liquid in the glass capillary and *g* is the gravitational acceleration.^21,22^5
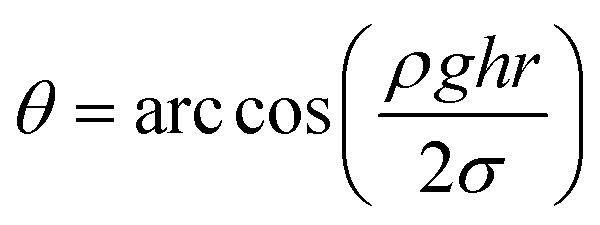


## 3. Results and discussion

### 3.1 FTIR spectrum

**Fig. 3:**
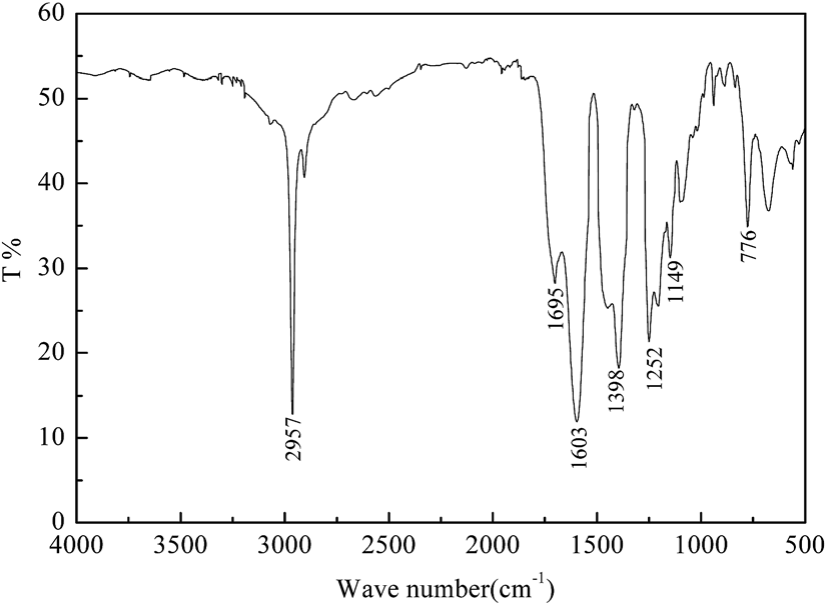
FTIR spectrum of the gas-wetting alteration agent.

The infrared spectrum of the gas-wetting alteration agent is shown in Fig. 3. The absorption peak of C–H appears at 2957 cm^−1^. The peaks at 1252 cm^−1^ and 1149 cm^−1^ correspond to the characteristic absorption of –CF_3_ and –CF_2_, respectively. The stretching vibration absorption of the C=O–N group occurs at 1695 cm^−1^. Moreover, the characteristic peak of the C=O–NH group at 3300–3100 cm^−1^ disappears, indicating that the monomers have reacted adequately. Furthermore, the carboxylate stretching peaks are shown at 1603 cm^−1^ and 1398 cm^−1^.

### 3.2 ^1^H NMR spectrum

**Fig. 4:**
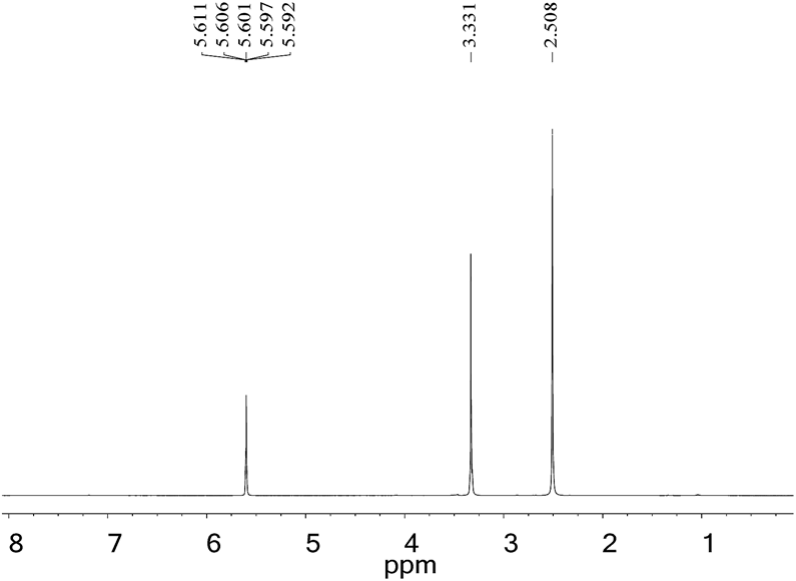
^1^H NMR spectrum of the gas-wetting alteration agent.

The gas-wetting alteration agent was characterized by ^1^H NMR and the spectrum is presented in Fig. 4. The peaks at *δ* 2.508 and *δ* 3.331 correspond to the solvent (DMSO) and deionized water, respectively. The single peak at *δ* 5.601 for H is assigned to the –CH_2_ group. This observation can be explained by the chemical shift of hydrogen in the methylene group moving upfield due to the strong electron-withdrawing amide group and the role of the carboxylate. The result is in good agreement with the infrared analysis, indicating that the product has been successfully synthesized.

### 3.3 Thermal stability

**Fig. 5:**
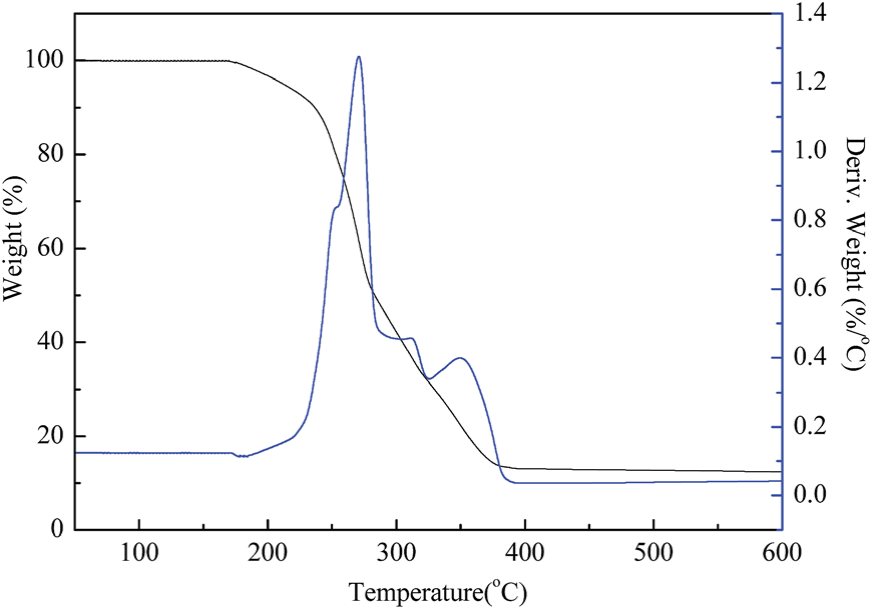
The thermal stability curves of the gas-wetting alteration agent.

The thermal analysis revealed the stability of the gas-wetting alteration agent when the temperature is increased. As seen in Fig. 5 there are four stages of weight loss in the TGA curve. During the first stage, before 170 °C, there is almost no weight loss. In the second stage, from 170 °C to 230 °C, the weight loss is about 9%, which may be attributed to the evaporation of the residual solvent during the purification process. There was a significant increase in weight loss between 230 °C and 400 °C, with the maximum weight loss rate peaking at about 1.267% °C^−1^ at 270 °C with a relatively narrow reaction interval. The acceleration of weight loss at this stage indicates that thermal decomposition has occurred. Above 400 °C, the remaining weight ratio is less than 10%, which can be assigned to the carbon residue. These results show that the synthesized gas-wetting alteration agent decomposes at higher temperatures compared to literature data (120–140 °C).^23,24^ This demonstrates that it has a great potential in terms of temperature tolerance and can be applied to unconventional reservoirs.

### 3.4 Contact angle measurements

**Fig. 6:**
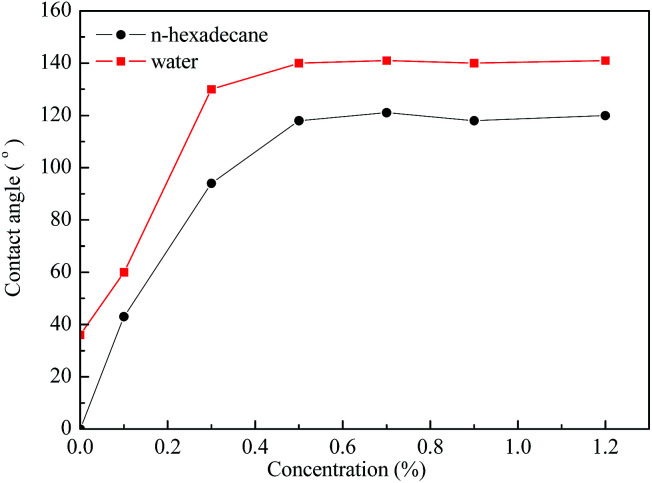
The contact angles of water and *n*-hexadecane on rock surfaces.

**Fig. 7:**
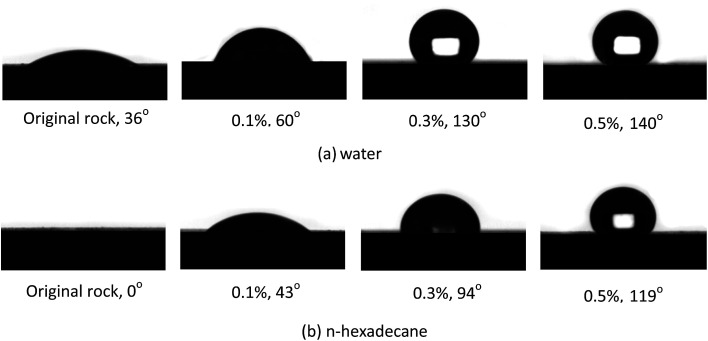
The pictures of the droplets on the rock suface.

The contact angles of water and *n*-hexadecane on the rock surface before and after treatment were measured and the results are shown in Fig. 6. Initially, the rock surface showed strong liquid-wetting with contact angles of water and *n*-hexadecane of 36° and 0°, respectively. However, after treatment with the gas-wetting alteration agent, the wettability of the rock was changed to preferential gas-wetting. When the concentration of the gas-wetting alteration agent was 0.1 wt%, the contact angle of water changed from 36° to 60°, while that of *n*-hexadecane increased from 0° to 43°. With the increase in concentration of the agent, the contact angles of water and *n*-hexadecane increased continuously. When the concentration was 0.5 wt%, the contact angles reached maximums of 140° and 119°, respectively. The results were better than the literature data.^25–27^ The respective contact angles of water and *n*-hexadecane remained at about 140° and 119° with further increases in concentration. Pictures of the drops on the rock surface before and after treatment with different concentrations are shown in Fig. 7.

This observation may be explained as follows: at low concentrations, a small amount of molecules are adsorbed on the surface of the rock through van der Waals forces and these adsorbed molecules are easily desorbed or replaced by molecules in other solutions. At higher concentrations, the adsorption capacity is greater than that of desorption, resulting in an increase of the amount of gas-wetting alteration agent adsorbed on the rock and the formation of a gas-wetting molecular layer on its surface. As a result of this, the wettability of the rock is altered from strong liquid-wetting to gas-wetting. With a further increase of concentration, the adsorption capacity of the rock surface tends to be saturated and so the wettability becomes stable. Therefore, the contact angle of water or *n*-hexadecane remains essentially unchanged.^28,29^

### 3.5 The surface free energy

The surface free energy is a crucial feature when altering the wettability of a rock surface. Table 2 shows the change in the surface free energy of the rock before and after treatment with different concentrations. It can be seen that the surface free energy was rapidly reduced from 72 mN m^−1^ to 3.4 mN m^−1^ after treatment with a concentration of 0.3 wt%. With the increase in concentration, the surface free energy did not decrease significantly. This result is consistent with studies by Gindl and Jiang *et al.*^30,31^ However, when the concentration exceeded 0.9 wt%, the surface free energy had a tendency to gradually increase. A reasonable explanation is that when the polar segments of the gas-wetting alteration agent are adsorbed into the rock by electrostatic forces, the non-polar segments with double-repellent chains are exposed to the surface of the rock. Moreover, these further form a strong hydrophobic and oleophobic molecular layer which alters the wettability of the rock surface. Unfortunately, when the concentration exceeds the optimum value, the surface free energy has a tendency to gradually increase with an increase in concentration. The reason is that the bimolecular chains may be entwined with each other, leading to a portion of the nonpolar segments being locked. As a result, the surface free energy exhibits a small rebound.^32^

**Table 2:** The surface free energy of the rocks

Concentration (%)	0	0.3	0.5	0.7	0.9	1.2
Surface free energy (mN m^−1^)	72	3.4	3.2	2.9	4.5	7.1

### 3.6 Imbibition

**Fig. 8:**
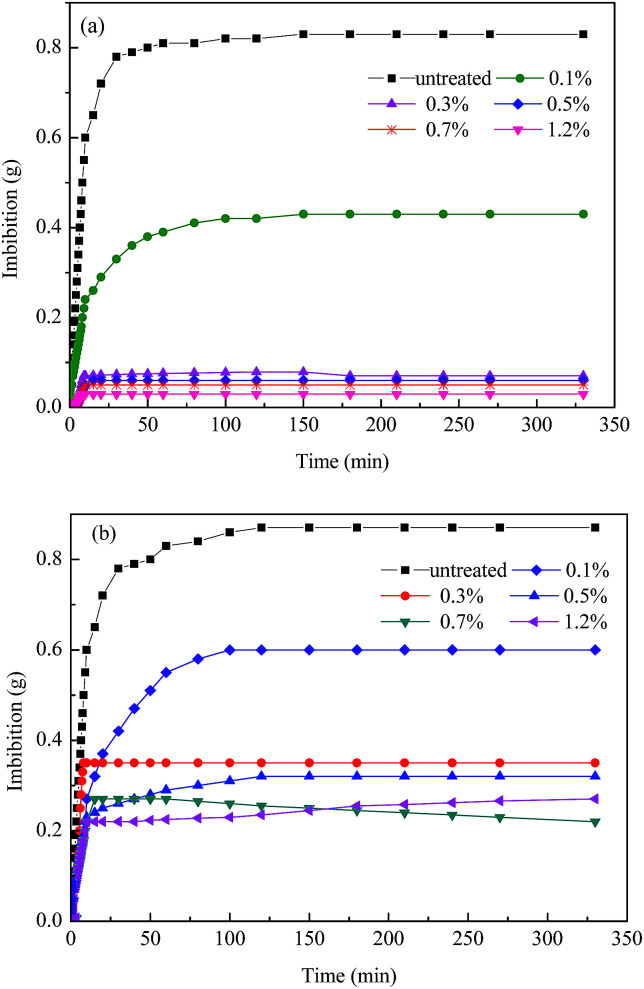
Imbibition by liquid into the rock before and after treatment: (a) water and (b) *n*-hexadecane.

The wettability of the rock before and after treatment was determined by spontaneous imbibition. The dynamic process of water or *n*-hexadecane imbibed into the rock was conducted by recording the weight of the liquid over time until there was no significant change. Fig. 8(a) shows that the volume of water imbibed into the rock was 0.83 g without the chemical treatment, while the value sharply decreased to 0.07 g after treatment with 0.3 wt%. This indicates that the rocks have become strongly hydrophobic. Fig. 8(b) shows the *n*-hexadecane imbibed into untreated and treated rock. The amount of imbibed rock decreased significantly from 0.87 g to 0.25 g after treatment, which demonstrates that the rock has an oleophobic property after chemical treatment. The substantial reduction in both water and *n*-hexadecane reveals that the wettability of the rock has been altered from strong liquid-wetting to gas-wetting, which agrees with the results of Giraldo *et al.*^33^ and Lai *et al.*^34^

### 3.7 Capillary tube rise testing

**Fig. 9:**
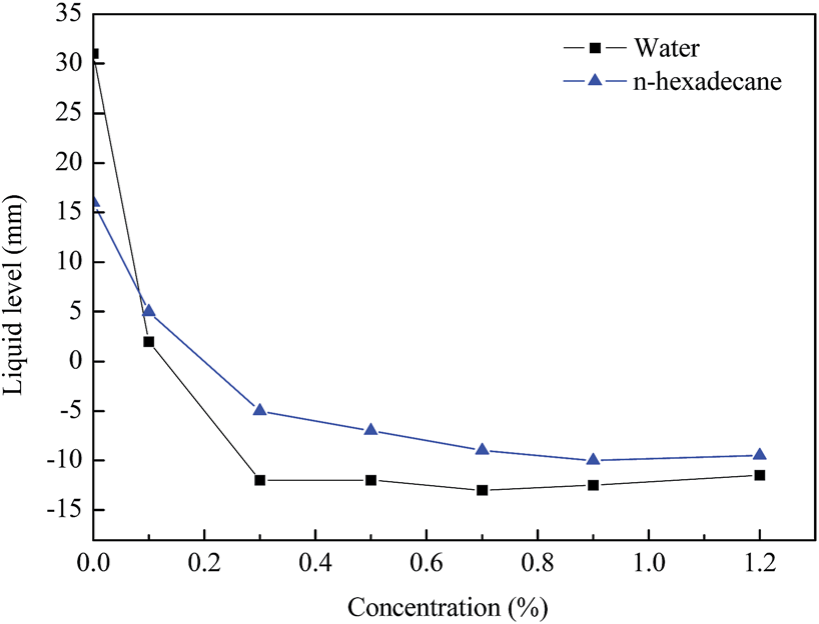
The water level in the capillaries before and after treatment.

The glass capillary tube rise test was performed to further demonstrate the wettability alteration of the rock. Fig. 9 summarizes the liquid level in the capillary tube as a function of the treatment concentrations. It can be clearly observed that the water level was rapidly reduced from 31 mm to approximately −13 mm as the concentration of the agent increased. Moreover, the *n*-hexadecane level was confirmed to show a similar trend to the water. Therefore, it was sufficient to indicate that the wettability of the capillary inner wall was changed to gas-wetting. The results of the glass capillary rise testing were consistent with literature data.^35^ Accordingly, the agent could effectively improve the rate of fracturing fluid flowback and maintain the blocks with high yields, while promoting the outflow of extra liquid to reduce any damage to the formation.

### 3.8 SEM

**Fig. 10:**
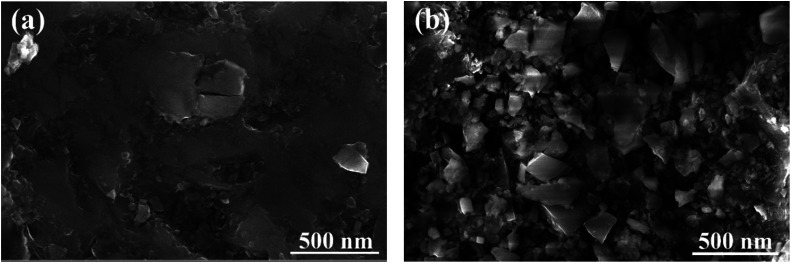
SEM images of the rock surface: (a) original rock and (b) treated.

Fig. 10 displays the microstructure of the rock surface before and after treatment. Fig. 10(a) shows that almost no material covered the surface of the original rock and its surface was relatively smooth. This observation could promote the expansion of liquid on the rock’s surface, which further illustrates the reason why the initial wettability of the reservoir is liquid-wetting. However, it can be clearly seen from Fig. 10(b) that the morphology of the rock surface has been significantly changed after treatment with the gas-wetting alteration agent. The surface of the rock adsorbs a large number of agent molecules through van der Waals forces and forms a tight adsorption layer, which effectively improves the roughness of the rock surface. In addition, the surface free energy can be significantly reduced after treatment. Thus, the wettability of the rock surface is altered from water-wetting or oil-wetting to gas-wetting. This is consistent with what has been reported in the literature.^36,37^

### 3.9 EDS

**Fig. 11:**
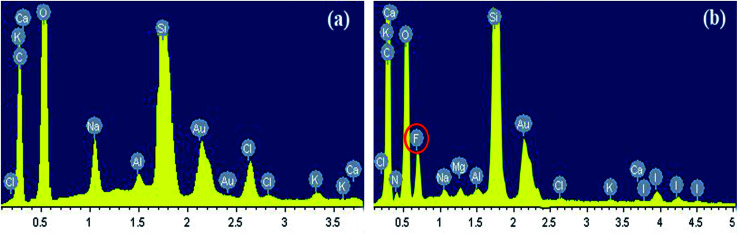
Results of EDS of the rock surface: (a) original rock and (b) treated.

Fig. 11 illustrates the elements of the rock surface before and after it was modified by the gas-wetting alteration agent. As shown in Fig. 11(a), there was no fluorine element in the original rock sample, which was mainly composed of elements such as calcium, potassium, oxygen and silicon. The presence of Au was due to the spray before the test. It can be seen from Fig. 11(b) that fluorine has been found on the treated rock surface. This shows that fluorine can be sufficiently adsorbed on the surface of the rock. This is the primary cause of the gas-wetting alteration of the reservoir and is consistent with the research of Aminnaji *et al.*^38^

## 4. Conclusions

In this work a gas-wetting alteration agent was synthesized and characterized by FTIR and ^1^H NMR. Thermal gravimetric analysis exhibited that the synthesized gas-wetting alteration agent can resist temperatures up to 230 °C. The contact angle method was used to examine the wettability of the rock before and after treatment. The results show that the wettability of the rock surface is altered from its original strong liquid-wetting to gas-wetting using 0.5 wt% of the agent. The results can be further verified by imbibition measurements and capillary tube rise testing. The wettability alteration of the reservoir surface can be explained by the special chemical structure, compared with the studies of Iglauer *et al.*^39^ and Sharma *et al.*^40^ The polar segments can be adsorbed into the rock by electrostatic forces, while the non-polar segments with double-repellent chains are exposed to its surface and form a strong hydrophobic and oleophobic molecular layer. Furthermore, the analysis by SEM and EDS also suggests that the effect of the agent on gas-wetting alteration can be ascribed to the increase in the roughness of the rock surface and the decrease in the surface free energy.

## Conflicts of interest

There is no conflict to declare.

## Supplementary Material
